# Characterisation of IncI1 plasmids associated with change of phage type in isolates of *Salmonella enterica* serovar Typhimurium

**DOI:** 10.1186/s12866-021-02151-z

**Published:** 2021-03-27

**Authors:** Lester Hiley, Rikki M. A. Graham, Amy V. Jennison

**Affiliations:** Public Health Microbiology, Queensland Reference Centre for Microbial and Public Health Genomics (MPHG), Forensic and Scientific Services, Queensland Department of Health, Coopers Plains, Queensland Australia

**Keywords:** *Salmonella* Typhimurium, IncI1 plasmid, Gene composition, Sequence comparison

## Abstract

**Background:**

Acquisition of IncI1 plasmids by members of the *Enterobacteriaceae* sometimes leads to transfer of antimicrobial resistance and colicinogeny as well as change of phage type in *Salmonella* Typhimurium. Isolates of *S.* Typhimurium from a 2015 outbreak of food poisoning were found to contain an IncI1 plasmid implicated in change of phage type from PT135a to U307 not previously reported. The origin of the changes of phage type associated with this IncI1 plasmid was investigated. In addition, a comparison of its gene composition with that of IncI1 plasmids found in local isolates of *S.* Typhimurium typed as U307 from other times was undertaken. This comparison was extended to IncI1 plasmids in isolates of phage types PT6 and PT6 var. 1 which are thought to be associated with acquisition of IncI1 plasmids.

**Results:**

Analysis of IncI1 plasmids from whole genome sequencing of isolates implicated a gene coding for a 1273 amino acid protein present only in U307 isolates as the likely source of change of phage type. The IncI1 plasmids from PT6 and PT6 var. 1 isolates all had the *ibfA* gene present in IncI1 plasmid R64. This gene inhibits growth of bacteriophage BF23 and was therefore the possible source of change of phage type. A fuller comparison of the genetic composition of IncI1 plasmids from U307 isolates and PT6 and PT6 var. 1 isolates along with two IncI1 plasmids from *S.* Typhimurium isolates not showing change of phage type was undertaken. Plasmids were classified as either ‘Delta’ or ‘Col’ IncI1 plasmids according to whether genes between *repZ* and the *rfsF* site showed high identity to genes in the same location in R64 or ColIb-P9 plasmids respectively. Comparison of the *tra* gene sets and the *pil* gene sets across the range of sequenced plasmids identified Delta and Col plasmids with almost identical sequences for both sets of genes. This indicated a genetic recombination event leading to a switch between Delta and Col gene sets at the *rfsF* site. Comparisons of other gene sets showing significant variation among the sequenced plasmids are reported. Searches of the NCBI GenBank database using DNA and protein sequences of interest from the sequenced plasmids identified global IncI1 plasmids with extensive regions showing 99 to 100% identity to some of the plasmids sequenced in this study indicating evidence for widespread distribution of these plasmids.

**Conclusion:**

Two genes possibly associated with change of phage type were identified in IncI1 plasmids. IncI1 plasmids were classified as either ‘Delta’ or ‘Col’ plasmids and other sequences of significant variation among these plasmids were identified. This study offers a new perspective on the understanding of the gene composition of IncI1 plasmids. The sequences of newly sequenced IncI1 plasmids could be compared against the regions of significant sequence variation identified in this study to understand better their overall gene composition and relatedness to other IncI1 plasmids in the databases.

**Supplementary Information:**

The online version contains supplementary material available at 10.1186/s12866-021-02151-z.

## Background

IncI plasmids are self-replicating double-stranded circular DNA elements which belong to the incompatibility (Inc) group IncI and are defined as plasmids producing type 1 pili susceptible to phage If1 [1]. There are three IncI variants, I1, Iγ and I2 [2]. The IncI1 variant is much more common than the other two. IncI1 plasmids occur widely in members of the *Enterobacteriaceae* and are present in many serovars of *Salmonella* including Typhimurium where their acquisition by conjugative transfer is sometimes implicated in change of phage type [3] and transfer of antimicrobial resistance [2].

Anderson and Lewis [4] reported on the occurrence of a transfer factor now known to be an IncI1 plasmid associated with antimicrobial resistance transfer in *Salmonella* Typhimurium (*S.* Typhimurium). Transfer of this factor was found to change the phage type of a PT36 strain sensitive to all 30 of the typing phages to PT6 which is sensitive to only 9 phages including phage 6. They called this plasmid a Delta resistance transfer factor. They showed that Delta plasmids could enter a strain of *Salmonella* or *Escherichia coli* (*E. coli*) containing a resistance factor located on the cell chromosome or possibly in the cytoplasm and then incorporate this R-factor into its own sequence. Mating experiments could then be performed to transfer resistance to a sensitive strain via the Delta plasmid containing the resistance factor. Anderson and Lewis [4] also identified related transfer factors which could attach chromosomal colicin genes and transfer colicinogeny to a new host strain. These were called ColI transfer factors.

Anderson et al. [3] provided more information about the typing phage patterns which distinguish between Delta and ColI plasmids. In a strain of *S.* Typhimurium sensitive to all the typing phages introduced Delta plasmids restricted many of the typing phages and produced a variety of phage patterns most commonly PT6 while ColI plasmids eliminated reaction only to closely related phages 12 and 13 thereby causing phage type conversion to PT125. ColI plasmids could carry colicin genes or they were absent but Delta plasmids almost never had colicin genes. They also identified other related plasmids which did not affect phage type.

Anderson et al. [3] proposed that the ColI transfer factors which caused phage type conversion to PT125 be designated gamma type transfer factors to distinguished them from Delta type factors. This is not acceptable now that the incompatibility group IncIγ has been created. For this reason, the ‘Col’ plasmid term will be applied in this report regardless of whether the plasmids carry colicin genes. The ‘Col plasmid’ term will be applied to any IncI plasmid which has any ColIb-P9-like genes from nt 1487–15,609 in ColIb-P9 GenBank Acc. No. NC_002122 following the *repZ* gene. The ‘Delta plasmid’ term will be applied to any IncI plasmid which has any R64-like genes from nt 29,968–43,619 in R64 GenBank Acc. No. AP005147 excluding antimicrobial or metals resistance genes.

*S.* Typhimurium plasmid R64 is a Delta plasmid [3] and has two genes which inhibit bacteriophage infection [5]. One is the *ibfA* gene (nt 32,653–34,209 GenBank Acc. No. AP005147) which inhibits T5 phages such as BF23 and the other is the *pifA* gene (nt 40,141–42,366 AP005147) which inhibits T7 phages. R64 has been shown also to inhibit a P22 phage in *S.* Typhimurium [6]. Since most of the Anderson typing phages are P22 phages it becomes clear why Delta plasmids can alter phage type. The ColIb-P9 plasmid from *Shigella sonnei* (*S. sonnei*) also has an *ibfA* gene (nt 13,401–13,745 GenBank Acc. No. NC_002122) which inhibits coliphages T5 and BF23 but it is unrelated to the *ibfA* gene in R64 [7]. The mechanisms by which these plasmids alter phage type have not been fully resolved.

The Public Health Microbiology Laboratory at Forensic and Scientific Services (Queensland Department of Health) receives all *Salmonella* isolates from laboratories in Queensland and Northern New South Wales for serotyping and for further genotyping for certain serovars. Serovar Typhimurium isolates have been routinely typed by phage typing methods from 1999 to 2011 after which phage typing has only been applied on a selective basis. From 2006 to the present all Typhimurium isolates have been genotyped by MLVA methods [8]. By the application of extended VNTR typing and CRISPR typing it has been shown that *S.* Typhimurium isolates can be assigned to Repeats Groups (RGs) [9] whose phylogenetic relationships have been determined by means of SNPs phylogeny methods [10].

In 2015, there was an outbreak of *S*. Typhimurium gastrointestinal illness involving several food outlets in the Brisbane, Australia, region with over 400 human isolates as well as food and environmental isolates (Additional file 1: Text S1). MLVA typing showed that most isolates had the same MLVA profile but there were another two single locus variants associated with isolates from another food outlet and environmental source. For all MLVA types most of the isolates submitted for phage typing were the U307 phage type with a few from two MLVA types identified as PT135a, a variant of DT135 [11] commonly found in Australia. Selected human and non-human isolates representing both phage types were subjected to whole genome sequencing. An IncI1 plasmid was identified in the U307 isolates but not in the PT135a isolates which had an almost identical chromosomal sequence to the U307 isolates, providing evidence that the plasmid was the agent of the change of phage type.

As a result of this finding we have undertaken an investigation into the genetic composition of the U307-associated plasmid to understand how this plasmid might affect phage type. Following on from the work of Anderson and Lewis [4] we have extended that investigation to include IncI1 plasmids associated with change of phage type to DT6 and DT6 var. 1 in three different genotypes of *S*. Typhimurium. We have further undertaken to make a broad-ranging comparison of the genetic composition of twenty-six plasmids sequenced for this study in order to understand more fully their relationships to each other and to other IncI plasmids in the NCBI database. This work has enabled us to discover IncI1 plasmids which are very closely related or almost identical to one of seven of the plasmids we have sequenced.

## Results and discussion

### Analysis of sequenced isolates from 2015 outbreak

The details of the 2015 outbreak of *S.* Typhimurium infection are provided in supplementary information (Additional file 1: Text S1). Eight outbreak isolates were chosen for sequencing: four patient isolates (three phage type U307 and one PT135a), encompassing three closely similar MLVA profiles, and four isolates (three U307 and one PT135a with two of the same MLVA profiles) from food and environmental sources (Table 1). As an outlier a patient isolate 15ST010303 (PT135a) which shared one of the MLVAs implicated in the outbreak but was isolated outside the outbreak period was included.

**Table 1:** Characteristics of sequenced isolates of *S*. Typhimurium from 2015 outbreak

Isolate ID	Source	Euro MLVA^b^	Phage Type	Isolation Date	Accession number
P024_15	Door handle	2–10-10-11-0212	135a	5/01/2015	ERR4159265
P212_15	Raw chicken	2–10-10-11-0212	U307	13/01/2015	ERS2213021
P581_15	Eggs	2–10-10-11-0212	U307	6/02/2015	ERR4159266
P216_15	Drag swab	2–10-9-11-0212	U307	14/01/2015	ERR4159267
15ST00430	Faeces	2–10-10-11-0212	135a	7/01/2015	ERR4159260
15ST00542	Faeces	2–10-10-11-0212	U307	10/01/2015	ERR4159261
15ST00840	Faeces	2–10-9-11-0212	U307	14/01/2015	ERR4159262
15ST01671	Faeces	2–10-10-12-0212	U307	27/01/2015	ERR4159263
15ST010303^a^	Faeces	2–10-10-12-0212	135a	13/11/2015	ERR4159264

^a^Outlier ^b^The European MLVA coding system

**Fig. 1:**
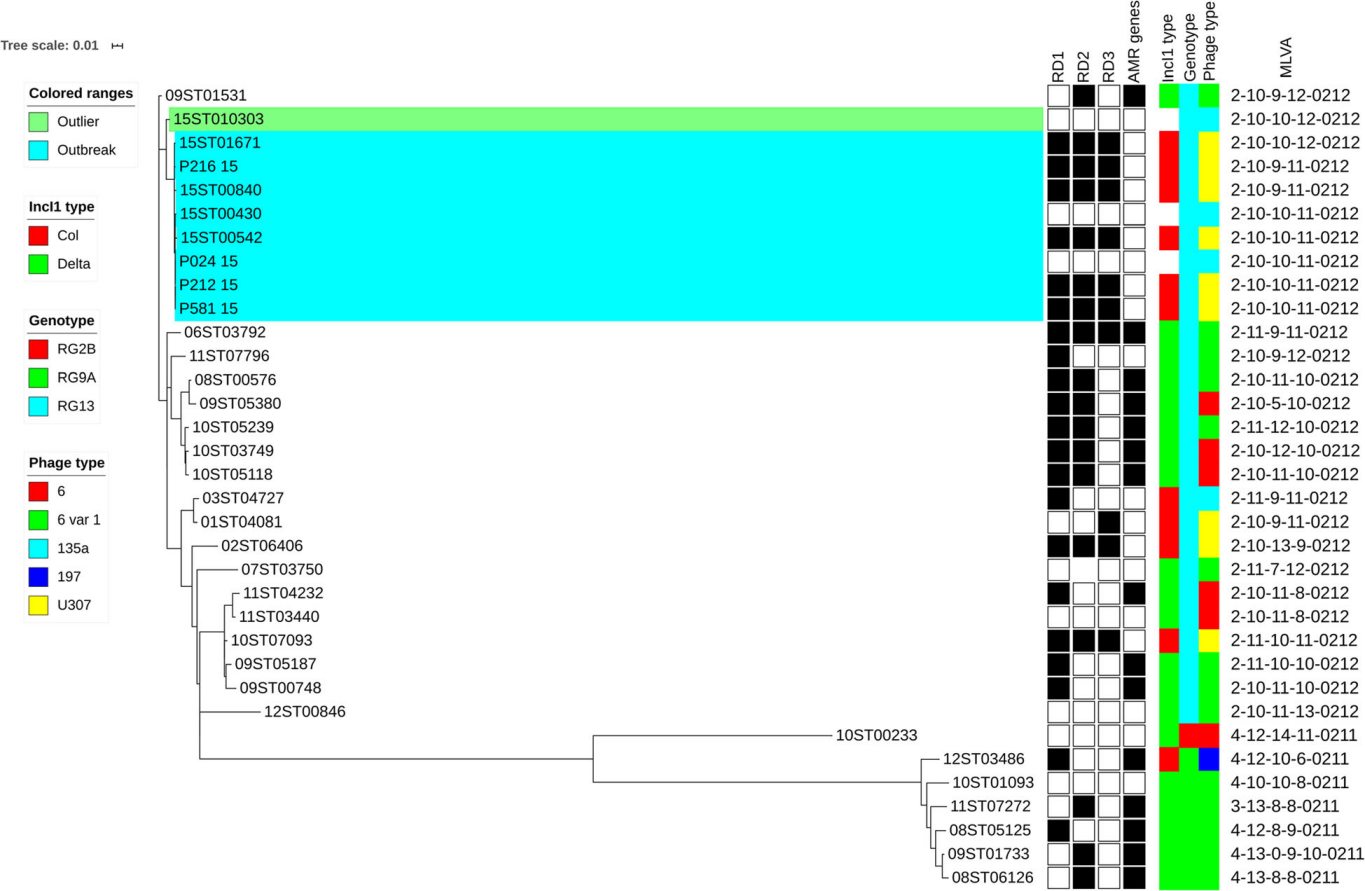
Maximum-likelihood phylogeny based on core chromosomal SNPs for 2015 outbreak isolates and for additional isolates of *S.* Typhimurium as listed in Table 3. The reference sequence is *S*. Typhimurium 01ST04081 (GenBank Acc. No. CP029840). The scale bar shows the proportion of total substitutions for the site. The legend shows the genotype, phage type, presence of antimicrobial resistance (AMR) genes and European MLVA for the isolates. The legend includes characteristics for the contained IncI1 plasmid when present in the isolate. RD1, RD2 and RD3 are regions of DNA sequence difference (see text) in the plasmids and the IncI1 type shows whether they are ‘Col’ or ‘Delta’ plasmids. No result for IncI1 type indicates absence of plasmid

Analysis of the outbreak isolates using core chromosomal SNPs with *S*. Typhimurium 01ST04081 (GenBank Acc. No. CP029840) as a reference showed that the outbreak isolates were genetically similar with one to seven SNPs difference across the two phage types and three MLVAs (Fig. 1). The outlier isolate (15ST010303) was 19 to 21 SNPs different from the outbreak isolates showing that it was genetically distinct from the outbreak strains. The files of assembled contigs for the outbreak isolates were BLASTed (https://blast.ncbi.nlm.nih.gov/Blast.cgi) against the sequence for *S.* Typhimurium LT2 and a variety of phage sequences found in *S.* Typhimurium [9]. All of the isolates belonged to the RG13 genotype [9] and all had the P2 prophage, P2-Hawk (nt 42,375–74,414 GenBank Acc. No. AMDY02000013) as well as the P4 prophage (nt 2,889,844–2,900,569 GenBank Acc. No. NC_016810) [12]. A plasmid sequence which was determined to belong to the plasmid incompatibility group IncI1 was identified in all of the isolates typed as U307 but not in the isolates typed as PT135a. This plasmid was further investigated as the likely agent of the phage type conversion from PT135a to U307.

The isolate P212_15 had a single contig 93,336 bp in size which was an almost intact IncI1 plasmid except for shufflon genes *pilVB* and *pilVC* (nt 130,208–104,220 R64 Acc. No. AP005147) which were located on other contigs. BLASTing of complete and draft IncI1 plasmid sequences in *Enterobacteriaceae* against the P212_15 IncI1 contig showed that there were three regions of DNA sequence in P212_15 IncI1 frequently missing from other IncI1 plasmids. Relative to R64 IncI1 sequence (AP005147) these were: a 1250 bp sequence containing a 361 aa protein called FinQ with fertility inhibition function which inserted just after a 173 bp intergenic sequence located at nt 74,801–74,973 in R64 between the *trbA* and *pndA* genes leading to duplication of the intergenic sequence at the RH flank of the inserted sequence; a 1819 bp sequence containing a 409 aa transposase protein for insertion sequence IS609, called InsQ, which inserted at nt 75,496 in R64; a 4165 bp sequence containing a gene for a 1273 aa protein which was substituted for the nt 34,658–43,604 sequence in R64. Henceforth these three regions of difference are referred to as RD1, RD2 and RD3 respectively.

### Source of phage type conversion in U307, PT6 and PT6 var. 1 isolates

In order to investigate a possible role for any of the three regions of difference in phage type conversion PCR primer pairs targeting the principle gene in all three regions were chosen (Additional file 2: Table S1) and PCR tests were developed. A panel of *S.* Typhimurium isolates from our collection of local isolates was assembled for testing (Additional file 3: Table S2). U307 isolates were chosen from years 2000 to 2011. PT135 and PT135a isolates from a similar time period were chosen because they were thought to be like U307 isolates without a plasmid. In addition PT6 and PT6 var. 1 isolates of different genotypes were selected because it was suspected that they would have IncI1 plasmids which may also be responsible for phage type change. Furthermore, three PT197 isolates belonging to one of the PT6 var. 1 genotypes, RG9A were included. It was of interest to look at the antimicrobial profiles of the chosen phage types because antimicrobial resistance is a common feature of IncI1 plasmids. A survey of local isolates of *S*. Typhimurium collected between 2006 and 2011 showed that thirteen out of 26 (50%) of PT6 isolates and 33 out of 38 (87%) of PT6 var. 1 isolates in our collection had antimicrobial resistance. In contrast, only one out of 79 U307 isolates, 30 out of 1270 (2.4%) PT135a isolates, 20 out of 494 (4%) PT135 isolates and 17 out of 408 (4%) PT197 isolates showed antimicrobial resistance.

Testing for the three regions of difference in the chosen panel of isolates showed that RD3 was present in all 44 U307 isolates in the panel and was exclusive to them (Additional file 3: Table S2). Nearly all these U307 isolates also had both RD1 and RD2 but three were missing one or both. Out of 63 PT135a and PT135 isolates only one had a region of difference, RD1. Twenty four out of 29 PT6 or PT6 var. 1 isolates were positive for one or both of RD1 and RD2. Even PT6 or PT6 var. 1 isolates negative for all three markers were thought to probably have an IncI1 plasmid because they had most likely undergone phage type conversion. One of the three PT197 isolates had a region of difference (RD1).

The correlation between the presence of RD3 and phage type U307 was evidence that the 1273 aa protein coded by the gene in RD3 was the possible agent of phage type conversion from PT135 or PT135a to U307. All of the U307 isolates belonged to genotype RG12D or RG13 [9]. Comparison of the phage patterns for PT135, PT135a and U307 showed that, relative to PT135, the PT135a pattern lacks reactivity for closely related phages 12 and 13 and has reduced reactivity for all of the phages reactive in the PT135 pattern (Table 2). Relative to the PT135a pattern, the U307 pattern lacks reactivity for seven phages (4, 5, 6, 14, 15, 27 and 35) and has reduced reactivity for one (25) but has increased reactivity for fourteen phages (2, 3, 10, 11, 16, 17, 19, 20, 21, 22, 23, 24, 26 and 32).

**Table 2:** *S.* Typhimurium phage typing patterns for PT135, PT135a, U307, PT6 and PT197. PT6 var. 1 pattern is reaction for phage 6 alone

	***Salmonella*** Typhimurium typing phage reactions																													
Phage	1	2	3	4	5	6	7	8	10	11	12	13	14	15	16	17	18	19	20	21	22	23	24	25	26	27	28	29	32	35
**135**	–	+	++	SCL	SCL	<CL	–	–	SCL	SCL	CL	CL	SCL	+++	CL	CL	–	+++	<SCL	<SCL	++	CL	**+++**	+++	++	CL	–	CL	+++	<CL
**135a**	–	5	+	++	+	++	–	–	++	+	–	–	++	++	+++	++	–	+	+	+	3	++	2	+++	2	4	–	CL.	5	++
**U307**	–	+++	+++	–	–	–	–	–	SCL	SCL	1	1	–	–	SCL	CL	–	+++	SCL	+++	++	CL	+++	+	+	–	–	CL	SCL	–
**6**	–	–	–	–	+	OL	–	–	_/+	_/+	–	–	–	–	_/ <CL	_/+	–	–	–	–	–	–	–	–	–	_/ +++	_	OL	_	_/ +++
**197**	SCL	_/+	SCL	CL	CL	CL	CL	–	CL	CL	+	+	CL	CL	<CL	<CL	–	CL	CL	CL	+	CL	CL	CL	++/< SCL	CL	–	CL	CL	SCL

*CL* Confluent lysis, *SCL* Semi-confluent lysis, *OL* Opaque lysis

+++ = > 100 plaques

+++ = 80–100 plaques

++ = 60–80 plaques

++ = 40–60 plaques

+ =20–40 plaques

+ = 6–20 plaques

1–5 plaques

To investigate the significance of the RD1 and RD2 sequences in PT6, PT6 var. 1 and other phage types a further 25 isolates representing a range of phage types, genotypes, antimicrobial resistance profiles and RD status were sequenced (Table 3).

**Table 3:** Sequenced isolates including P212/15 isolate from 2015 outbreak showing characteristics of the contained IncI1 plasmids

Isolate ID	Phage Type	Euro MLVA	Genotype	AR Profile	Type of IncI1	RD Status*	Accession Number
01ST04081	U307	2–10-9-11-0212	RG13	TET TRI	Col	3	ERR2309154
02ST06406	U307	2–10-13-9-0212	RG13	Nil	Col	1 + 2 + 3	ERR4159247
03ST04727	135a	2–11-9-11-0212	RG13	Nil	Col	1	ERR4159248
06ST03792	6 var. 1	2–11-9-11-0212	RG13	TET SUL KAN	Delta	1	ERR4159249
07ST03750	6 var. 1	2–11-7-12-0212	RG13	Nil	Delta	2	ERR4159248
08ST00576	6 var. 1	2–10-11-10-0212	RG13	STR TET	Delta	1 + 2	ERR2309155
09ST00748	6 var. 1	2–10-11-10-0212	RG13	TET KAN	Delta	1	ERR2309157
09ST05187	6 var. 1	2–11-10-10-0212	RG13	TET	Delta	1	ERR4159251
09ST05380	6	2–10-5-10-0212	RG13	STR TET CHL SUL	Delta	1 + 2	ERR4159252
10ST03749	6	2–10-12-10-0212	RG13	STR	Delta	1 + 2	ERS2213017
10ST05118	6	2–10-11-10-0212	RG13	STR TET	Delta	1 + 2	ERR4159253
10ST05239	6 var. 1	2–11-12-10-0212	RG13	STR TET	Delta	1 + 2	ERR4159254
10ST07093	U307	2–11-10-11-0212	RG13	Nil	Col	1 + 2 + 3	ERR2309164
11ST03440	6	2–10-11-8-0212	RG13	Nil	Delta	Nil	ERR2309165
11ST07796	6 var. 1	2–10-9-12-0212	RG13	Nil	Delta	1	ERR4159255
12ST00846	6 var. 1	2–10-11-13-0212	RG13	AMP	Delta	Nil	ERR4159256
P212_15	U307	2–10-10-11-0212	RG13	Nil	Col	1 + 2 + 3	ERS2213021
11ST04232	6	2–10-11-8-0212	RG13	STR	Delta	1	ERR2309166
10ST00233	6	4–12-14-11-0211	RG2B	Nil	Delta	Nil	ERR2309161
09ST01531	6 var. 1	2–10-9-12-0212	RG13	AMP SPC	Delta	2	ERR2309158
10ST01093	6 var. 1	4–10-10-8-0211	RG9A	AMP	Delta	Nil	ERR2309162
08ST05125	6 var. 1	4–12-8-9-0211	RG9A	SUL TRI	Delta	1	ERR4159257
09ST01733	6 var. 1	4–13–0-9-10-0211	RG9A	AMP	Delta	2	ERR2309159
08ST06126	6 var. 1	4–13-8-8-0211	RG9A	AMP	Delta	2	ERR2309156
11ST07272	6 var. 1	3–13-8-8-0211	RG9A	AMP SPC	Delta	2	ERR4159258
12ST03486	197	4–12-10-6-0211	RG9A	AMP	Col	1	ERR4159259

*RD Region of Difference in plasmid (See text)

The phylogenetic tree generated from the SNPs analysis of the outbreak isolates and the additional sequenced isolates shows the spread of genotypes and the relationship of the additional isolates to the outbreak isolates (Fig. 1). The RG2B genotype and the RG9A genotypes were well separated from each other and from the rest of the isolates which were RG13 genotype [10]. The 2015 outbreak isolates fell into a tight cluster separate from most of the other RG13 isolates. The profiles for the IncI1 plasmids in the isolates are shown in Fig. 1. Note that 15ST010303, 15ST00430 and P024_15 lack plasmid.

Sequence data was assembled into contigs and plasmid sequences were identified by alignment against reference IncI1 plasmids ColIb-P9 (NC_002122) and R64 (AP005147). Large contigs and plasmid assemblies were subjected to gene determination and annotation by RAST (https://rast.nmpdr.org/). It was found that all of the PT6 and PT6 var. 1 isolates had the *ibfA* gene present in R64 but four were missing the *pifA* gene in R64. None of the plasmids from other phage types had either of the R64 *ibfA* or *pifA* genes. This was evidence that the IbfA protein may also inhibit P22 typing phages and may be the agent of phage type conversion to PT6 and PT6 var. 1.

Sequenced PT6 and/or PT6 var. 1 isolates came from three genotypes of *S.* Typhimurium, RG2, RG9A and RG13 (Table 3) [10]. Phage type conversion for the RG9A isolates from PT197 to PT6 var. 1 occurs through loss of reactivity of all typing phages except phage 6 (Table 2). Phage type conversion for the RG13 isolates from PT135 or PT135a to PT6 or PT6 var. 1 also occurs through loss of reactivity to many of the same typing phages leaving reactivity to up to nine phages including phage 6 for the PT6 pattern and to phage 6 only for PT6 var. 1. Since all of the typing phages are related to P22 except for phages 12 and 13 [13] it follows that plasmids in the PT6 and PT6 var. 1 isolates have inhibited P22 phages but in a different pattern from the plasmid in U307 isolates which inhibits phage 6. Phage 6 was derived from the propagating strain for phage 13 and is not related genealogically to any of the other typing phages [13].

The plasmids in two isolates, 03ST04727 (PT135a) and 12ST03486 (PT197), did not cause phage type conversion. Both plasmids were identified as Col plasmids. The plasmid from 03ST04727 (genotype RG13 and RD1 only) had a full set of ColIb-P9-specific genes including the ColIb-P9-specific *ibfA* gene which inhibits coliphages T5 and BF23. If it had entered a PT36 strain it should have caused phage type conversion to PT125 by inhibiting typing phages 12 and 13 [3]. However, the phage type of 03ST04727 would have already been PT135a which has inhibition of phages 12 and 13 in its phage pattern (Table 2) so no phage type conversion would be seen. The plasmid from 12ST03486 (genotype RG9A and RD1 only) had some ColIb-P9-specific genes but was missing all such genes after *yagA* including *ibfA.* The PT197 phage pattern includes reaction to phages 12 and 13 (Table 2) so it was evident that the plasmid in 12ST03486 had not caused inhibition of these phages. This may be because it is missing the *IbfA* gene. It is therefore possible that the *ibfA* gene in ColIb-P9 is the agent of phage type conversion by Col plasmids observed by Anderson et al. 1973 [3].

### Comparative DNA sequence analysis of sequenced IncI1 plasmids

**Fig. 2:**
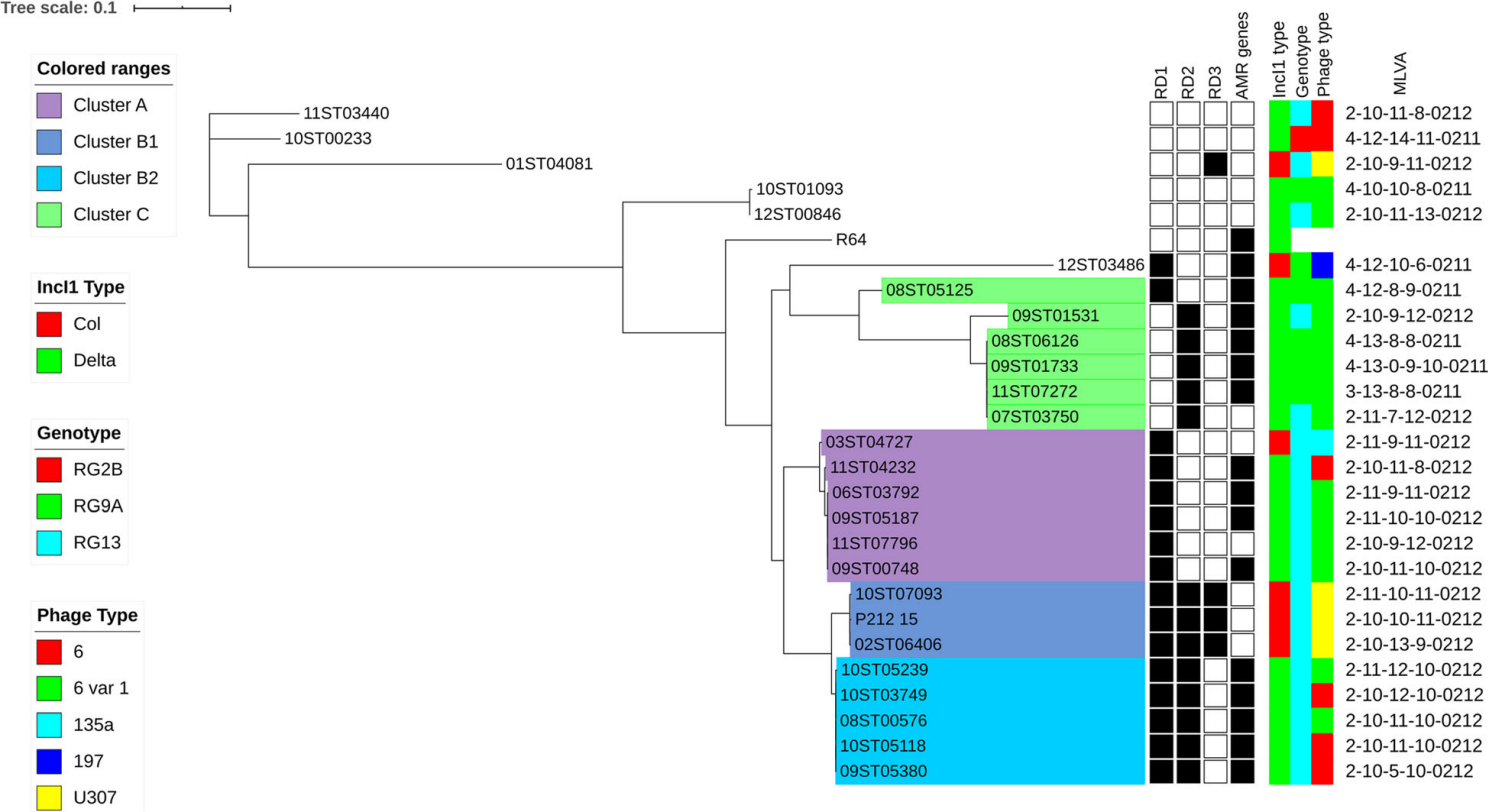
Maximum-likelihood phylogeny based on core SNPs for IncI1 plasmids from isolates of *S.* Typhimurium. The reference sequence is IncI1 plasmid R64 (GenBank Acc. No. AP005147). The scale bar shows the proportion of total substitutions for the site. The legend shows the genotype, phage type, presence of antimicrobial resistance (AMR) genes and European MLVA for the isolates. The legend includes characteristics for the contained IncI1 plasmid when present in the isolate. RD1, RD2 and RD3 are regions of DNA sequence difference (see text) in the plasmids and the IncI1 type shows whether they are ‘Col’ or ‘Delta’ plasmids

As a first approach towards understanding the structure of the sequenced IncI1 plasmids (Table 3) an analysis based on core SNPs was performed in order to compare the sequences of shared gene sets. This process excludes sequences belonging to the accessory genome as well as sequences which distinguish Delta plasmids from Col plasmids. It also excludes sequences which are common to most sequenced plasmids but which have been deleted from at least one of them. An example of this was seen in 09ST05380 which was missing 9177 bp extending from the *rfsF* site to *yfcA* gene common to most Delta and Col IncI1 plasmids. The resulting phylogenetic tree (Fig. 2) showed that there were three main clusters with closely related shared gene sets. Cluster A consisted of six plasmids which all had RD1 only. Four were from PT6 var. 1 isolates, one was from a PT6 isolate (11ST04232) and one from a PT135a isolate (03ST04727). BLASTing against Delta gene sequences from R64 and the Col gene sequences from ColIb-P9 showed that the plasmids from the PT6 var. 1 and PT6 isolates had high identity to sequences in the Delta gene set. The plasmid from the PT6 isolate 11ST04232 was missing the sequence from *ydfB* to just before the *rfsF* site, including the *pifA* gene, deleted by insertion of a large antimicrobial and metals resistance sequence. The plasmid from the PT135a isolate 03ST04727 had almost 100% identity to the Col gene set. This showed that plasmids with related shared gene sets could be either Delta or Col plasmids and that a switch between Delta and Col gene sets had occurred [5].

Cluster B consisted of two subsets. Cluster B1 consisted of plasmids from three U307 isolates, P212_15 from the 2015 outbreak and two isolates from years 2002 and 2010. All had all three RD sequences and Col-specific gene sets truncated by the substitution of Col genes by the RD3 sequence within the *ydeA* gene and just before the *rfsF* site. Cluster B2 consisted of plasmids from five PT6 or PT6 var. 1 isolates all having RD1 plus RD2 and all with Delta-specific gene sets. The plasmid from one PT6 isolate 09ST05380 was missing the *pifA* gene. The observation that the two subsets, B1 and B2, have similar shared gene sets suggests that this may be another example of a switch between Delta and Col gene sets. All of the Cluster A and Cluster B plasmids came from genotype RG13 isolates.

Cluster C consisted of plasmids from six PT6 var. 1 isolates, all with Delta-specific gene sets. Five had RD2 only while the plasmid from 08ST05125 with a more distantly related shared gene set had RD1 only. Two were from genotype RG13 hosts (07ST03750 and 09ST01531) and four including the 08ST05125 plasmid were from genotype RG9A hosts showing that there could be movement of closely related plasmids between host genotypes.

The last six plasmids consisted of: two Delta plasmids with closely related shared gene sets and with no RD sequences, one from a genotype RG9A PT6 var. 1 host (10ST01093) and the other from a genotype RG13 PT6 var. 1 host (12ST00846); two Delta plasmids with more distantly related shared gene sets and no RD sequences and no *pifA* gene, one from a genotype RG2B PT6 host (10ST00233) and the other from a genotype RG13 PT6 host (11ST03440); a Col plasmid with RD1 only from a genotype RG9A PT197 host (12ST03486) and a Col plasmid with RD3 only from a genotype RG13 U307 host (01ST04081).

### Further analysis of sequenced IncI1 plasmids

The next approach towards understanding the structure of these plasmids was firstly to make a closer examination of the shared gene components, specifically the *tra* and *pil* gene sets from the transfer region, to find out how they compared with each other. This would be followed by a comparison of those sequences excluded from the comparison of shared gene sets in plasmids, namely the Delta and Col gene sets, sequences from *trbA* to *traY* which traverses the region where RD1 and RD2 sequences are located and the *rfsF* to *yfcA* sequence missing from 09ST05380.

#### Comparison of *tra* and *pil* gene sets in plasmids of sequenced isolates

IncI1 plasmids produce two kinds of sex pili, a thin flexible pilus coded by the *pil* genes and a thick rigid pilus coded by the *tra* genes [14]. The clusters in the phylogenetic tree are largely a product of the *tra* and *pil* gene sets since so many genes are excluded from the core SNPs analysis because they are not shared by all of the plasmids. The *tra* gene sets in the sequenced plasmids were BLASTed against sequences extending from the *excA* gene to the shufflon gene, *rci,* in both R64 and ColIb-P9. The *pil* gene sets were BLASTed against the sequence from the shufflon gene to *traD* in R64 and from the shufflon gene to *trcD* in ColIb-P9.

All of the plasmids in Cluster A (Fig. 2) had almost identical *tra* gene sets. They had 99% identity with the R64 and ColIb-P9 sets except for the *excA* and *traY* genes which had only 76 and 86% identity respectively with the genes in R64 and ColIb-P9 as well as some sequences with no identity in both genes. The *excA* and *traY* genes in the Cluster A plasmids were almost identical to those genes in R621a plasmid which belongs to the incompatibility group Iγ and has a different entry exclusion system from R64 and ColIb-P9 [15]. However, Cluster A plasmids all had the R64 *inc* sequence and not the R621a *inc* sequence and so belong to incompatibility group IncI1. The *tra* genes set in Cluster A plasmids had 98 to 99% identity to the set in R621a (excluding two insertion sequences in R621a) but was still different by 374 SNPs.

The *pil* gene set in all Cluster A plasmids was very close in identity to the same set in ColIb-P9. Like the ColIb-P9 set it was missing the *pilVD* gene, it had the *pilI, pilJ* and *pilK* genes like ColIb-P9 rather than those in R64 and had the *trcD* gene in ColIb-P9 instead of the *traD* gene in R64. Like ColIb-P9 it also had a 60 bp insert, not in R64, located directly after the *pilJ* gene but in all instances the Cluster A *pil* gene sets had a 9 bp tandem repeat (TR), not found in ColIb-P9, included in the inserted sequence. All the plasmids in Cluster A were Delta plasmids except for 03ST04727 plasmid which had a full set of Col genes. The host strain of this plasmid was phage type 135a and had therefore not undergone phage type conversion unlike the hosts of the Delta plasmids. The Col plasmid had almost identical sets of transfer genes to the Delta plasmids in the cluster indicative of the switch between Col and Delta gene sets at the *rfsF* site [5] as noted previously.

The three plasmids in Cluster B1 from U307 hosts (Fig. 2) all had *tra* and *pil* gene sets very close in identity to those in ColIb-P9 and almost the same as both sets in the five plasmids belonging to Cluster B2 which closely aligned with Cluster B1. The *pil* gene sets in Cluster B1 plasmids, as in ColIb-P9, did not have the 9 bp TR in the 60 bp insert but it was present in the *pil* gene sets in Cluster B2 plasmids. Since the plasmids in Cluster B1 were all Col plasmids while the plasmids in Cluster B2 were all Delta plasmids this was another example of an apparent switch between Col and Delta gene sets at the *rfsF* site [5].

The six plasmids in Cluster C (Fig. 2) were all Delta IncI1 plasmids. The *tra* gene sets in the five plasmids with RD2 only were nearly the same and were in close identity to the set in ColIb-P9. They were about 85 SNPs different from the ColIb-P9 set and 50 SNPs different from the sets in Cluster B1 and Cluster B2 plasmids. The *pil* gene sets for the five RD2 only plasmids were also nearly the same. They were different from the *pil* gene sets in Cluster B1 and Cluster B2 plasmids. They had the full set of *pilV* genes (including *pilVD*) but did not have the 60 bp insert present in ColIb-P9. The *pilK* gene was in close identity to that in ColIb-P9 not that in R64 whereas the *pilI* and *pilJ* genes were nearly identical to those in R64 not ColIb-P9. They had the *traD* gene of R64 rather than the *trcD* gene of ColIb-P9. The *pilL* gene was 98% identity to both R64 and ColIb-P9 genes. In addition, there was a 282 bp insert coding for another gene between the *pilL* and *pilK* genes in all Cluster C plasmids.

The plasmid in 08ST05128 in Cluster C had RD1 only and was separated somewhat from other members of Cluster C (Fig. 2). It had almost the same *tra* gene set as in Cluster A plasmids. However it had the same *pil* gene set as in Cluster C plasmids with RD2 only except that it lacked the *pilVD* gene. Sampei et al. [5] have hypothesised that there is a recombination point between *excA* and *pilJK*. The 08ST05125 plasmid may have been the result of recombination between a plasmid with the *tra* gene set of Cluster A and another with the *pil* gene set of the RD2 only plasmids in Cluster C. This suggests that the recombination site is therefore close to the shufflon gene, *rci,* which is located between the *tra* and *pil* gene sets.

The plasmid with RD1 only from 12ST03486 PT197 was a Col IncI1 plasmid which had not caused phage type conversion. It had a full *tra* gene set which was 161 SNPs different from the *tra* gene set in ColIb-P9 and 257 SNPs different from the *tra* gene set in R64. The *excA* gene was the same as in ColIb-P9 although the *traY* gene differed somewhat from *traY* in both ColIb-P9 and R64. The *pil* gene set had all of the ColIb-P9 genes including the *trcD* gene and the 60 bp insert but also had the *pilVD* gene lacking in ColIb-P9.

The remaining five plasmids from isolates 12ST00846, 10ST01093, 01ST04081, 10ST00233 and 11ST03440 all had *tra* gene sets with *excA* and *traY* genes which had a combined identity of 82% with 59 or 60 gaps compared with the genes in R64 and ColIb-P9. The *excA* and *traY* genes in these plasmids were significantly different from those in Cluster A plasmids as well as all the other sequenced plasmids in this study. The protein sequence identity was about 67% or less to ExcA proteins and 91% or less to TraY proteins respectively compared with the other sequenced plasmids (detailed later in this report under the title “Comparison of the sequences from *trbA* to *traY* in representative plasmids”). A search of the NCBI database has identified many other IncI1 plasmids with the identical or nearly identical *excA* and *traY* genes to those in these five sequenced plasmids.

The two plasmids from isolates 12ST00846 and 10ST01093 which had none of the three RDs located very closely in the phylogenetic tree and were almost identical for almost all of their length after discounting for two IS26 sequences in 12ST00846. The *tra* gene sets had 98% or 99% identity to the sets in ColIb-P9 and R64 although there were 93 fewer SNPs relative to the R64 set. In addition to the differences in the *excA* and *traY* genes there were some differences in the *sogL* gene. The *pil* gene sets were almost identical to that from ColIb-P9 except for the presence of the *pilVD* gene and the 9 bp TR in the 60 bp insert in the two sequenced plasmids.

The plasmids from isolates 01ST04081, 11ST03440 and 10ST00233 had *tra* gene sets very similar to plasmids from isolates 12ST00846 and 10ST01093. There were about 30 fewer SNPs relative to the ColIb-P9 *tra* gene set. The set from 01ST04081 was interrupted by a 2553 bp insert between *traU* and *traT* consisting of three genes including a mobile element gene, and the set from 10ST00233 was missing the shufflon gene, *rci.* The three plasmids from 01ST04081, 11ST03440 and 10ST00233 had very similar *pil* gene sets which were significantly different from the sets in all of the other sequenced plasmids in this study. The overall identity to ColIb-P9 and R64 sets was about 95% with 25 gaps. The majority of the sequence differences were in the *pilS* and *pilR* genes (86% identity with 19 gaps). There was the same 282 bp insert between *pilL* and *pilK* as seen in the RD2 only plasmids in Cluster C and the *pilK* and *pilI* genes were in closer identity to those in ColIb-P9 than to those in R64. The *pilJ* gene was replaced by two small genes and there was no *traD* or *trcD* gene present. All of the *pilV* genes were present. A search of the NCBI database showed that there are many other IncI1 plasmids with a *pil* gene set similar to that in these three plasmids.

#### Comparison of Delta and Col gene sets in representative plasmids

**Fig. 3:**
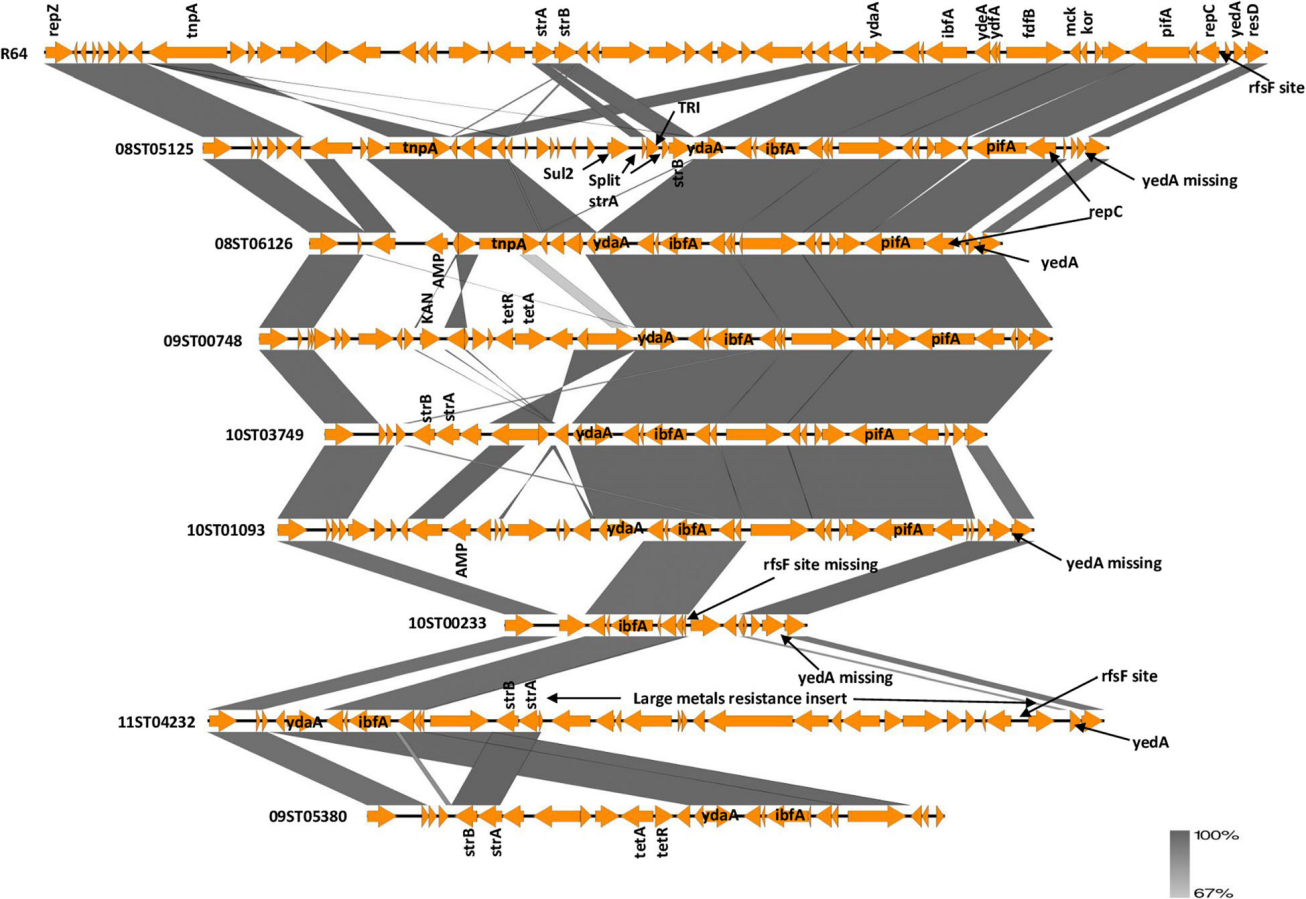
Comparison of Delta gene set sequences with left and right flanks for eight plasmids from PT6 or PT6 var 1 *S.* Typhimurium hosts with reference to plasmid R64. Gene names other than inserted genes are derived from R64 GenBank Acc. No. AP005147 annotation. Sul2 = sulphonamide resistance (Acc. No EBE3547935) TRI = trimethoprim resistance (Acc. No. WP_015058990.1) AMP = ampicillin resistance (Acc. No. AMM70781.1) KAN = kanamycin resistance (Acc. No. EAZ2053794.1) tetA and tetR = tetracycline resistance (Acc. No. EFN5607575.1 and ECL9996609.1). The bar indicates the level of relatedness. The length of the shafts is proportionate to the length of the gene and direction of the arrow heads indicates which strand the gene is located on. Drawn by Easyfig application [16]

A diagrammatical comparison of the Delta gene sets from eight representative plasmids from PT6 or PT6 var. 1 hosts with reference to plasmid R64 is shown in Fig. 3. The left flanking *repZ* gene and right flanking genes after the *rfsF* site are included to give context. Overall there was a high degree of variation in gene composition among these sets with a variety of gene insertions and deletions although a basic gene order was maintained. All eight plasmids had the inhibition of bacteriophage BF23 gene *ibfA* possibly associated with phage type conversion but three (10ST00233, 11ST04232 and 09ST05380) were missing the phage T7 exclusion protein gene *pifA* which does not appear therefore to be involved in phage type conversion. All except 10ST00233 had antimicrobial resistance genes as inserts into their gene sets usually in accord with their antimicrobial resistance profiles (Table 3). The exceptions were: 08ST05125 which had streptomycin resistance genes not shown in the resistance profile although the *strA* gene may have been inactivated by insertion of a trimethoprim resistance gene; 01ST04081 where the tetracycline and trimethoprim resistance genes were located on the pSLT plasmid rather than on the IncI1 plasmid; and 09ST05380 which had resistance to chloramphenicol and sulphonamide in its profile not present in the Delta gene set but these antimicrobial genes appeared to be located on an unrelated plasmid in this strain.

**Fig. 4:**
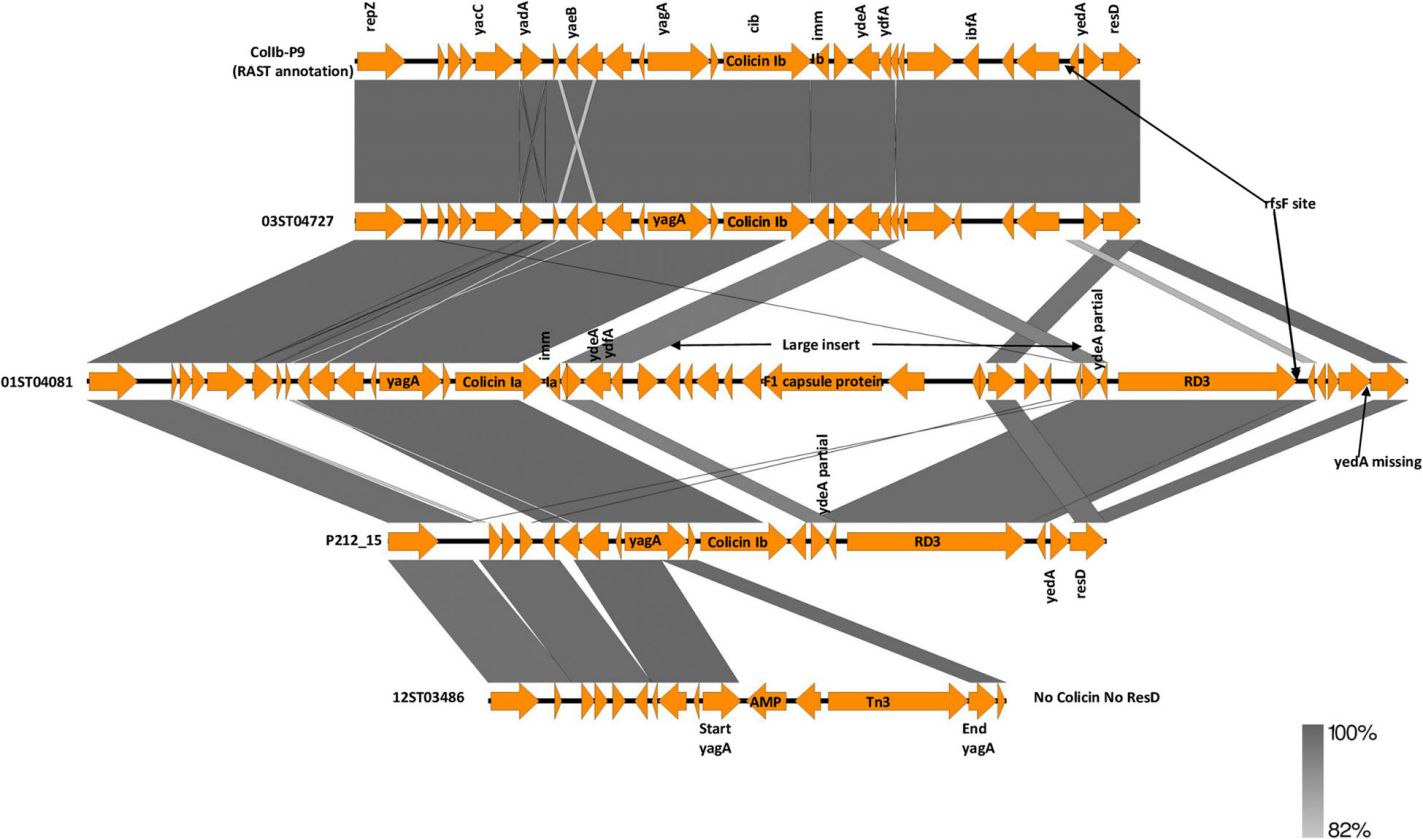
Comparison of Col gene set sequences with left and right flanks for four plasmids from U307, PT135a or PT197 *S.* Typhimurium hosts with reference to plasmid ColIb-P9. Gene names other than inserted genes were derived from ColIb-P9 GenBank Acc. No. NC_002122 annotation. The genes for ColIb-P9 were derived from a RAST version of the ColIb-P9 sequence different from the GenBank version. The bar indicates the level of relatedness. The length of the shafts is proportionate to the length of the gene and direction of the arrow heads indicates which strand the gene is located on. Drawn by Easyfig application [16]

The Col gene sets from four plasmids from two U307 hosts (01ST04081 and P212_15), a PT135a host (03ST04727) and a PT197 host (12ST03486) with reference to ColIb-P9 are shown in Fig. 4. The left flanking *repZ* gene and right flanking genes after the *rfsF* site are included to give context. The plasmid from 03ST04727 had very close identity to ColIb-P9. The plasmid from 01ST04081 had two large inserts including the RD3 sequence, which substituted for a number of genes from the ColIb-P9 gene set. It also had a colicin Ia gene and corresponding Ia immunity gene. The plasmid from P212_15 also had the RD3 sequence as well as three substitute genes replacing all the genes between *repZ* and *yaeB* in ColIb-P9 including *yacC* and *yadA*. Lastly, the plasmid from 12ST03486 started like P212_15 with the same substitute genes, had an ampicillin resistance gene cassette inserted into the *yagA* gene and was truncated after that so lacked any colicin gene as well as *resD*.

#### Comparison of the sequences from *trbA* to *traY* in representative plasmids

**Fig. 5:**
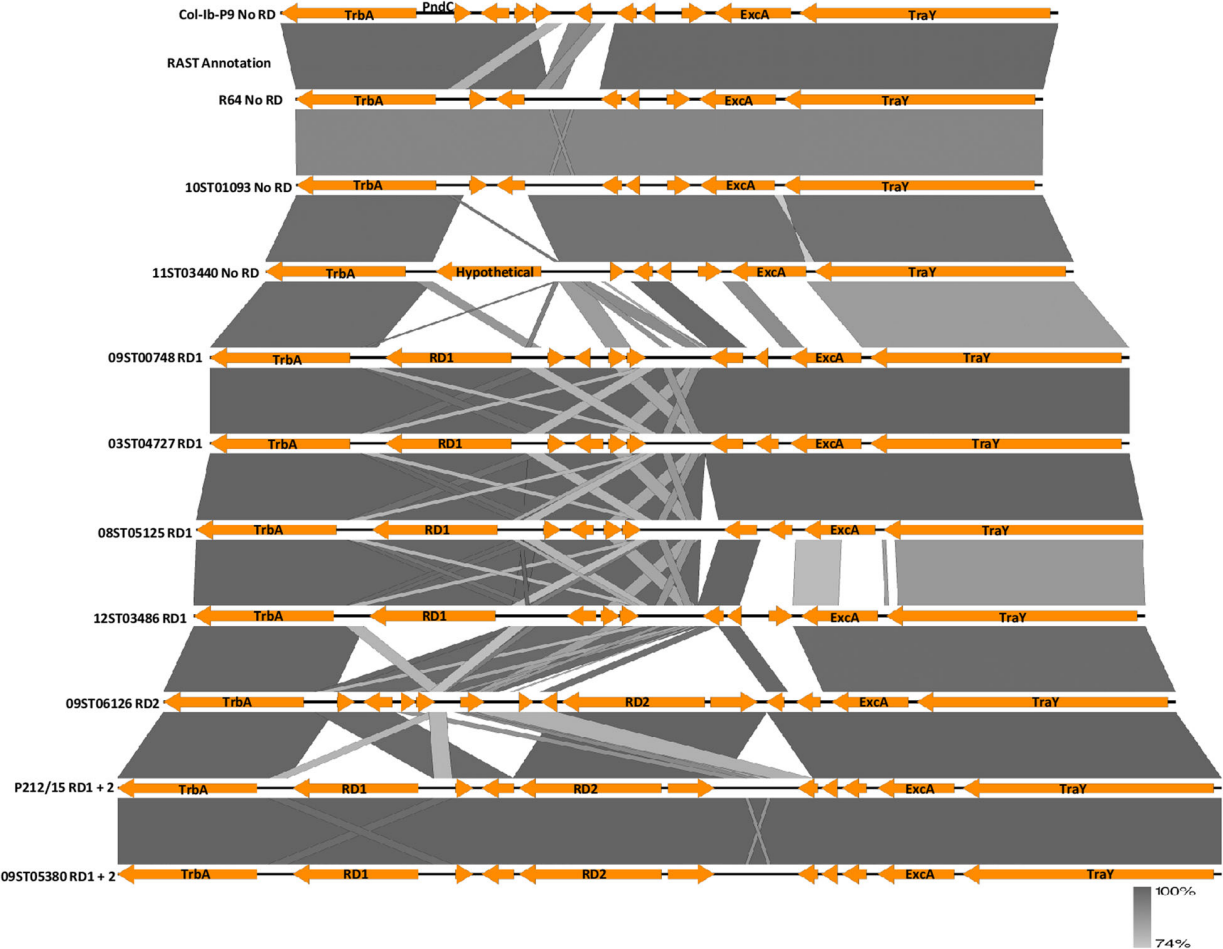
Comparison of representative IncI1 plasmid sequences from *trbA* to *traY*. Gene names other than inserted genes are derived from R64 GenBank Acc. No. AP005147 annotation. The bar indicates the level of relatedness. The length of the shafts is proportionate to the length of the gene and direction of the arrow heads indicates which strand the gene is located on. Drawn by Easyfig application [16]

This is a very variable region because it is where RD1 and RD2 sequences are located. A diagrammatical comparison of eleven strains is shown in Fig. 5. There was very little sequence difference for the *trbA* gene across all eleven strains. Note that this was true for 10ST01093 as well. The less than 100% shading for 10ST01093 compared with R64 in the figure was related to averaging of the %identity across the whole sequence. Most of the reduced identity was located in the *excA* and *traY* genes which averaged 82% identity with 59 gaps. All the isolates in Cluster C excluding 08ST05125 had the same sequence as 08ST06126. 10ST00233 was the same as 11ST03440. 12ST03486 was nearly the same (21 SNPs difference) as ColIb-P9 but had the RD1 insert.

The considerable sequence variation among the *excA* and *traY* genes from the sequenced strains (Fig. 5) was examined. Searches in the NCBI database using BLASTp and the protein sequences coded by *excA* and *traY* identified further variants giving a total of six distinct variants of *excA* and nine distinct variants of *traY*. The relationships to one another of the variants of *excA* and *traY* genes demonstrated by phylogenetic trees (Additional file 4: Fig. S1 and Additional file 5: Fig. S2 respectively) as well as alignments for the protein sequences for the variants of the *excA* and *traY* genes (Additional file 6: Fig. S3 and Additional file 7: Fig. S4 respectively) are presented in Supplementary information.

Variants of ExcA were used in BLASTp searches against the NCBI database and IncI1 plasmids in representative strains were examined to see what variant(s) of TraY accompanies each variant of ExcA and also whether the plasmid is a Delta or Col IncI1 plasmid by aligning it against the plasmid sequences for R64 and ColIb-P9 which have complete sets of Delta and Col genes respectively (Additional file 8: Text S2).

#### Comparison of the *rfsF* to *yfcA* 9177 bp sequences in plasmids of sequenced isolates

09ST05380 had all sequence missing between *ydJA* and *yfcA.* Consequently, the genes from the *rfsF* site to *yfcA* which are shared by Delta and Col IncI plasmids have not been included in the data used to generate the phylogenetic tree. We have compared this set of genes in the sequenced isolates by BLASTing with the R64 sequence nt 43,620–52,796 which is almost the same as in ColIb-P9. The results are presented in Additional file 9: Text S3.

### Variation in the *ydaA* gene from Delta plasmids

*ydaA* is the gene just near the start of the Delta gene set at nt 30,418–31,551 in R64 (AP005147). It was noticed that this gene can show variation in nucleotide sequence compared with R64 most notably with some members of Cluster A, Cluster B2 and some plasmids with no RD. The six Cluster C plasmids had the same *ydaA* variant as R64. Cluster A plasmids 09ST05187, 11ST07796 and 09ST00748 had a *ydaA* gene 94 SNPs different from the R64 gene. However the *ydaA* in 06ST03792 also in Cluster A had 17 SNPs relative to R64 and the *ydaA* in 11ST04232 also in Cluster A was the same as R64. Cluster B2 had all members with a *ydaA* gene like 09ST05187, 11ST07796 and 09ST00748 in Cluster A but with a 54 bp deletion between nt 508 and 561. The *ydaA* gene in 12ST00486 and 10ST01093 plasmids with no RD was like R64 but with an 88 bp deletion between nt 512 and 599 and 4 SNPs. The *ydaA* gene was missing from the 10ST00233 and 11ST03340 plasmids. The results of searches for YdaA protein variants in the NCBI database are presented in Additional file 10: Text S4.

### Identification of IncI1 plasmids with extensive or almost total identity with sequenced plasmids in this study

Through the use of BLASTp and/or BLASTn searches against fully sequenced IncI1 plasmids or plasmid sequences contained in draft genomes in the NCBI database a number of plasmids were identified which have 85 to 98% sequence cover and 99 to 100% sequence identity to one of seven of the IncI1 plasmids in this study (Table 4). The matches relate mostly to the IncI1-specific sequences but sometimes to the accessory gene sequences as well. The list of matching plasmids found is not always complete especially for 10ST00233 which has numbers of close matches not shown. These results give insight into how extensively these plasmids may be spreading around the globe.

**Table 4:** GenBank Acc. Nos. and host characteristics for IncI1 plasmids which have high sequence cover and 99 to 100% sequence identity to one of the listed IncI1 plasmids from this study

Sequenced IncI1 plasmid	IncI1 plasmid NCBI	Plasmid Host/Source/Country/Year
08ST05125	ADJW01000035 RQPT01000050	*E. coli* H383/USA? *S.* Infantis/chicken/Australia/2005
10ST00233	JYXO01000014 ASRF01000099 LEMB01000025 MLBA01000019 & 48	*S.* Muenchen/turkey/USA/2011 *S.* Infantis/stool/Israel/2008 *E. coli*/urine/USA *E. coli*/chicken/India/2015
P212_15	FZIL01000016 AFQH01000079 & 80	*E.* coli/pig/Australia/2007 *E. coli* H494/UK?
09ST00748	ADTK0100000000^a^	*E. coli* MS 84–1/gut/USA
11ST04232	MYXP01000015 MXEC01000018	*S. enterica*/USA *S.* Typhimurium/pork/USA/2011
01ST04081	JYEE0100000000^a^ MOLA0100000000^a^	*E. coli*/chicken/Denmark/2009 *E. coli*/chicken/China/2015
08ST06126	AEVZ0100000000^a^ QOGE0100000000^a^	*E. coli* M919/USA? *E. coli* AVC162/poultry/Australia/2010

^a^IncI1 sequence found in several or more contigs

## Conclusion

This study of IncI1 plasmids of *S.* Typhimurium has produced evidence that two different types of IncI1 plasmids are associated with phage type conversion. One type consists of Col plasmids which carry a 4165 bp gene which substitutes for a number of ColIb-P9 genes from within the *ydeA* gene (not annotated in GenBank Acc. No. NC_002122) to the *rfsF* site. This 4165 bp gene possibly affects the reactivity of the P22 typing phages causing phage type conversion from PT135a to U307. The other type consists of Delta plasmids which carry the *ibfA* gene found in plasmid R64 AP005147. This *ibfA* gene possibly affects the reactivity of the P22 typing phages in a different way leading to phage type conversion from PT135a and PT197 to PT6 or PT6 var. 1. The comparison of the sequences of the IncI1 plasmids in this study indicates possible value in differentiating between IncI1 plasmids according to whether genes between *repZ* and the *rfsF* site make them Delta or Col IncI1 plasmids as originally proposed by Anderson et al. 1973 [3]. Antimicrobial resistance appears to be more likely in Delta than in Col plasmids. Determination of the type of *excA* and *traY* genes in the *tra* gene set as well as the gene composition of the accompanying *pil* gene set may provide clues to the possible origins of IncI1 plasmids in sequenced isolates and also facilitate discovery of closely related IncI1 plasmids in the database.

## Methods

### Bacterial strains and sequencing methods

For outbreak isolates (Table 1) and additional local isolates (Table 3) DNA was extracted from cultures grown overnight at 37 °C on horse blood agar, using the QiaSymphony DSP DNA Mini kit (Qiagen) according to the manufacturer’s protocol. DNA was prepared for sequencing using the Nextera XT kit (Illumina) and sequenced on the NextSeq500 using the NextSeq 500 Mid Output v2 kit (300 cycles) (Illumina) according to the manufacturer’s instructions.

For investigation into correlation between the three regions of difference and phage type *S.* Typhimurium isolates representing phage types U307, 135, 135a, 6 and 6 var. 1 for a range of genotypes were selected from our collection of locally isolated *Salmonella* strains (Additional file 3: Table S2).

### PCR procedures

For PCR amplification of the three regions of difference the primers in Additional file 2: Table S1 were used. The mastermix contained 2 mM MgCl_2_, 5 pmol each of F and R primer (Geneworks, Adelaide, South Australia) and 0.5 U of AmpliTaq Gold (Applied Biosystems, Foster City, Calif.); the initial cycling step was 94 °C for 10 min followed by 30 cycles of 94 °C for 30 s, 58.5 °C for 45 s and 72 °C for 1 m 30 s with a final 72 °C for 10 min; a 6 μl aliquot from each PCR tube was electrophoresed in a 1.5% agarose gel containing 0.5 μg/ml ethidium bromide at 120 V for 40 min.

### Bioinformatic analysis

Sequences generated were quality trimmed using Trimmomatic v0.36 [17]. Chromosomal core SNPs were determined by mapping reads to the *S*. Typhimurium LT2 chromosome (Acc. No. NC_003197.2) using the Snippy v4.2 pipeline (https://github.com/tseemann/snippy), with the default parameters. Core SNPs were aligned and used to generate a maximum likelihood tree using the RaxML wrapper in Geneious R10 (Biomatters, New Zealand), using the GTR CAT model and 100 bootstrap replicates [18]. Snippy 4.2 was used to identify SNPs in the plasmid sequences using the *S*. Typhimurium plasmid R64 (Acc. No. AP005147) as a reference, and running snippy-core to generate a full length alignment which was used to generate a maximum likelihood tree using the RaxML wrapper in Geneious R10 (Biomatters, New Zealand), using the GTR CAT model and 100 bootstrap replicates [18]. Sequences were de novo assembled into contigs using the SPAdes v3.10.1 assembler [19].

### Comparison of plasmids

IncI1 plasmid contigs were identified by BLASTing against sequences for R64 (Acc. No. AP005147) and ColIb-P9 (Acc. No. NC_002122) and were assembled into provisional plasmid sequences for many of the isolates. These assemblies and longer contigs for other isolates were subjected to gene identification and annotated using RAST (https://rast.nmpdr.org/). BLASTn and Megablast were used for alignment of DNA sequences and BLASTp for alignment of protein sequences (https://blast.ncbi.nlm.nih.gov/Blast.cgi). EasyFig [16] was applied to RAST generated annotations for comparison of gene sequences.

### Phage typing and antimicrobial resistance profiling

Isolates were sent to the Microbiological Diagnostics Unit, University of Melbourne, Australia for phage typing by the Anderson scheme and antimicrobial sensitivity testing.

## Supplementary Information

**Additional file 1. Text S1.** The 2015 Salmonella Typhimurium U307 outbreak.

**Additional file 2. Table S1.** Primer sequences used for detection by PCR of regions of difference RD1, RD2 and RD3 in IncI1 plasmids.

**Additional file 3. Table S2.** Characteristics of local isolates assembled for testing for three regions of difference

**Additional file 4. Figure S1.**Maximum-likelihood phylogeny of excA gene sequences from six IncI1 plasmids.

**Additional file 5. Figure S2.** Maximum-likelihood phylogeny of traY gene sequences from nine IncI1 plasmids.

**Additional file 6. Figure S3.** Alignment of ExcA protein sequences from six IncI1 plasmids.

**Additional file 7. Figure S4.** Alignment of TraY protein sequences from nine IncI1 plasmids.

**Additional file 8. Text S2.** Search results for variants of ExcA proteins in the NCBI database.

**Additional file 9. Text S3.** Search results for the rfsF to yfcA 9177 bp sequences in plasmids of sequenced isolates in the NCBI database.

**Additional file 10. Text S4.** Search results for variants of YdaA proteins in the NCBI database.

## Data Availability

Raw sequence files and associated metadata have been submitted to the European Nucleotide Archive with project accession number PRJEB25063.

## Abbreviations

Inc.: Incompatibility group
*S.* Typhimurium: *Salmonella* Typhimurium
*E. coli*: *Escherichia coli*
*S. sonnei*: *Shigella sonnei*
PT: Phage type
MLVA: Multilocus VNTR Analysis
VNTR: Variable number tandem repeat
CRISPR: Clustered regularly interspaced short palindromic repeats
RG: Repeats group
Acc. No.: Accession number
SNP: Single nucleotide polymorphism
AMR: Antimicrobial resistance
TR: Tandem repeat
RD: Region of difference
PCR: Polymerase chain reaction
