# Supplementary material for: Characterisation of IncI1 plasmids associated with change of phage type in isolates of Salmonella enterica serovar Typhimurium
Source: BMC Microbiol. 2021 Mar 27;21:92. doi: 10.1186/s12866-021-02151-z (PMC8004404; doi:10.1186/s12866-021-02151-z)
Supplement: Supplementary file 1 — Additional file 1. Text S1. The 2015 Salmonella Typhimurium U307 outbreak. [file 12866_2021_2151_MOESM1_ESM.doc]

**The 2015 *Salmonella* Typhimurium phage type U307 outbreak.**

Between November 2014 and April 2015 there was a series of outbreaks of food poisoning involving eight different food outlets. Associated with seven of these outlets were 436 human isolates, 43 isolates from food and environmental samples with the Euro MLVA profile 2-10-10-11-0212. From the eighth outlet there were twelve human isolates and another six from food or environmental samples with the Euro MLVA 2-10-11-11-0212. Prior to this period there had been only 20 isolates (five typed as PT135a) with the 2-10-10-11-0212 profile and six isolates (three before 2010 typed as DT6 var 1 or indeterminate and three in 2014 typed as U307) with the 2-10-11-11-0212 profile. Nearly all of the food and environmental isolates were phage typed as U307 with one 2-10-10-11-0212 isolate and two 2-10-11-11-0212 isolates typed as PT135a. Of the twelve human isolates phage typed there were four isolates from each profile typed as U307 and two from each profile as PT135a. Antimicrobial resistance profiles were done at the same time as phage typing but none of the U307 or PT135a types showed any resistance.

Genotyping tests applied to 44 isolates with MLVA 2-10-10-11-0212 and four isolates with MLVA 2-10-11-11-0212 showed that they all had an STTR7 allele of 380bp and the PT135a P2 and P4 prophage combination characteristic of PT135a isolates [1] and they therefore belonged to the RG13 genotype [2].

1. Hawkey J, Edwards DJ, Dimovski K, Hiley L, Billman-Jacobe H, Hogg G, et al. Evidence of microevolution of Salmonella Typhimurium during a series of egg-associated outbreaks linked to a single chicken farm. BMC Genomics. 2013;14:800. doi: 10.1186/1471-2164-14-800. PubMed PMID: 24245509; PubMed Central PMCID: PMCPMC3870983.

2. Hiley L, Fang NX, Micalizzi GR, Bates J. Distribution of Gifsy-3 and of variants of ST64B and Gifsy-1 prophages amongst Salmonella enterica Serovar Typhimurium isolates: evidence that combinations of prophages promote clonality. PLoS One. 2014;9(1):e86203. doi: 10.1371/journal.pone.0086203. PubMed PMID: 24475087; PubMed Central PMCID: PMCPMC3901673.
