# Supplementary material for: Characterisation of IncI1 plasmids associated with change of phage type in isolates of Salmonella enterica serovar Typhimurium
Source: BMC Microbiol. 2021 Mar 27;21:92. doi: 10.1186/s12866-021-02151-z (PMC8004404; doi:10.1186/s12866-021-02151-z)
Supplement: Supplementary file 2 — Additional file 2. Table S1. Primer sequences used for detection by PCR of regions of difference RD1, RD2 and RD3 in IncI1 plasmids. [file 12866_2021_2151_MOESM2_ESM.docx]

**SI Table. Primer sequences used for detection by PCR of regions of difference RD1, RD2 and RD3 in IncI1 plasmids**

| **Primer Name** | **Sequence (5’- 3’)** | **Target** | **Source** |
| --- | --- | --- | --- |
| U307 plasmid RD1-F | CCTGTCCAGTATGTATGCCAGTGT | RD1 (FinQ) | P212_15 contig 15 |
| U307 plasmid RD1-R | TCGTGAAGGACTTCTTGACTCCAG |  |  |
| U307 plasmid RD2-F | CTTCGTGACTCAGGAGGGGGATTC | RD2 (InsQ) | P212_15 contig 15 |
| U307 plasmid RD2-R | CATAAAGTCACAACGACCGTCAGC |  |  |
| U307 plasmid RD3-F | CGCTCAATCTTCATTCCCGGAGGG | RD3 | P212_15 contig 15 |
| U307 plasmid RD3-R | TTTGCTCGGGTGTGCGTTCTTCTG |  |  |
