# Supplementary material for: Characterisation of IncI1 plasmids associated with change of phage type in isolates of Salmonella enterica serovar Typhimurium
Source: BMC Microbiol. 2021 Mar 27;21:92. doi: 10.1186/s12866-021-02151-z (PMC8004404; doi:10.1186/s12866-021-02151-z)
Supplement: Supplementary file 3 — Additional file 3. Table S2. Characteristics of local isolates assembled for testing for three regions of difference [file 12866_2021_2151_MOESM3_ESM.docx]

**S2 Table. Characteristics of local isolates assembled for testing for three regions of difference**

| **Phage Type** | **No. isolates** | **Genotype** | **RD1** | **RD2** | **RD3** | **Antmicrobial resistance** |
| --- | --- | --- | --- | --- | --- | --- |
| U307 | 37 | RG13 | + | + | + | Nil |
| U307 | 4 | RG12D | + | + | + | Nil |
| U307 | 2 | RG13 | - | - | + | Nil |
| U307 | 1 | RG13 | - | + | + | Nil |
| 135a | 53 | RG13 | - | - | - | Nil |
| 135/135a | 7 | RG12C | - | - | - | Nil |
| 135a | 1 | RG13 | + | - | - | Nil |
| 135a | 2 | RG13 | - | - | - | AMP/AMP SUL |
| 6 | 5 | RG13 | + | + | - | STR TET |
| 6 | 3 | RG13 | - | - | - | Nil |
| 6 | 1 | RG13 | + | - | - | STR |
| 6 | 1 | RG9A | - | - | - | Nil |
| 6 | 1 | RG9A | - | + | - | AMP SPC |
| 6 | 1 | RG2 | - | - | - | Nil |
| 6 var 1 | 6 | RG13 | + | + | - | STR TET |
| 6 var 1 | 5 | RG13 | + | - | - | TET/TET KAN/TET SUL KAN/AMP TRI |
| 6 var 1 | 1 | RG13 | - | + | - | AMP SPC |
| 6 var 1 | 1 | RG13 | - | - | - | Nil |
| 6 var 1 | 1 | RG13 | - | + | - | Nil |
| 6 var 1 | 1 | RG9A | - | - | - | AMP |
| 6 var 1 | 3 | RG9A | - | + | - | AMP |
| 6 var 1 | 1 | RG9A | + | - | - | SUL TRI |
| 197 | 1 | RG9A | - | - | - | AMP STR |
| 197 | 1 | RG9A | - | - | - | SUL TRI |
| 197 | 1 | RG9A | + | - | - | AMP |
