# Supplementary figures and images for: Characterisation of IncI1 plasmids associated with change of phage type in isolates of Salmonella enterica serovar Typhimurium
Source: BMC Microbiol. 2021 Mar 27;21:92. doi: 10.1186/s12866-021-02151-z (PMC8004404; doi:10.1186/s12866-021-02151-z)

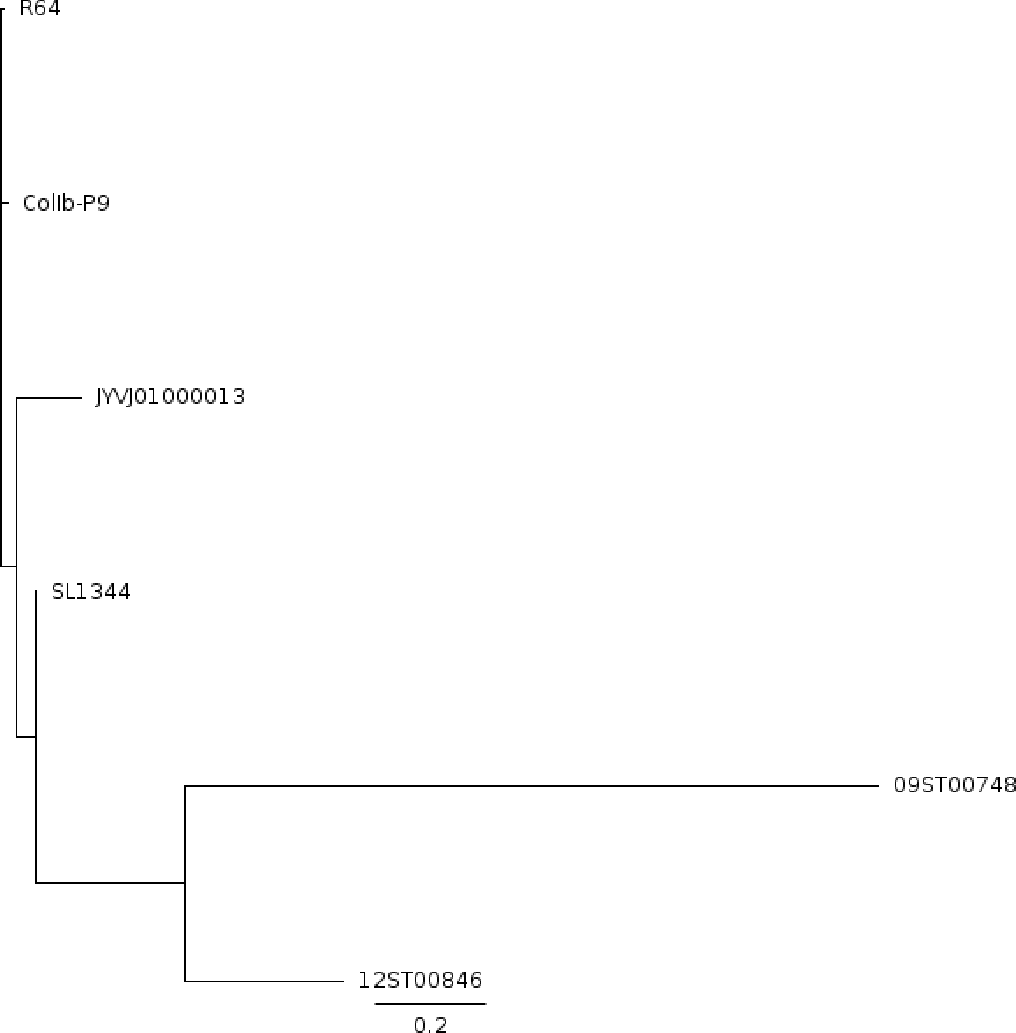

Supplement: Supplementary file 4 — Additional file 4. Figure S1.Maximum-likelihood phylogeny of excA gene sequences from six IncI1 plasmids. [file 12866_2021_2151_MOESM4_ESM.tif]

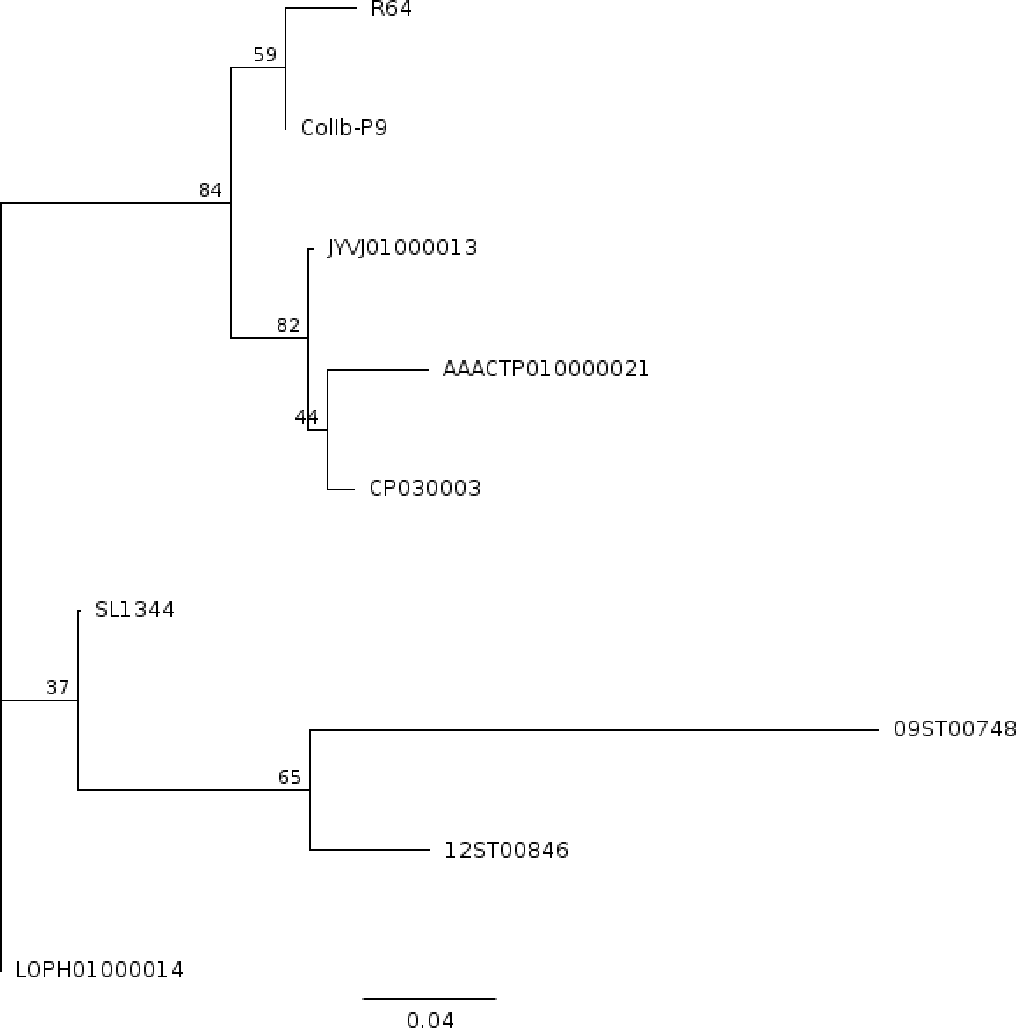

Supplement: Supplementary file 5 — Additional file 5. Figure S2. Maximum-likelihood phylogeny of traY gene sequences from nine IncI1 plasmids. [file 12866_2021_2151_MOESM5_ESM.tif]

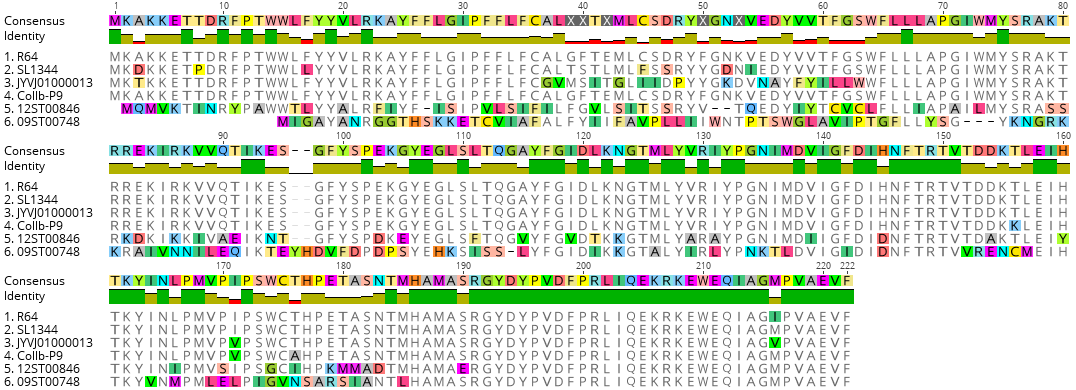

Supplement: Supplementary file 6 — Additional file 6. Figure S3. Alignment of ExcA protein sequences from six IncI1 plasmids. [file 12866_2021_2151_MOESM6_ESM.tif]

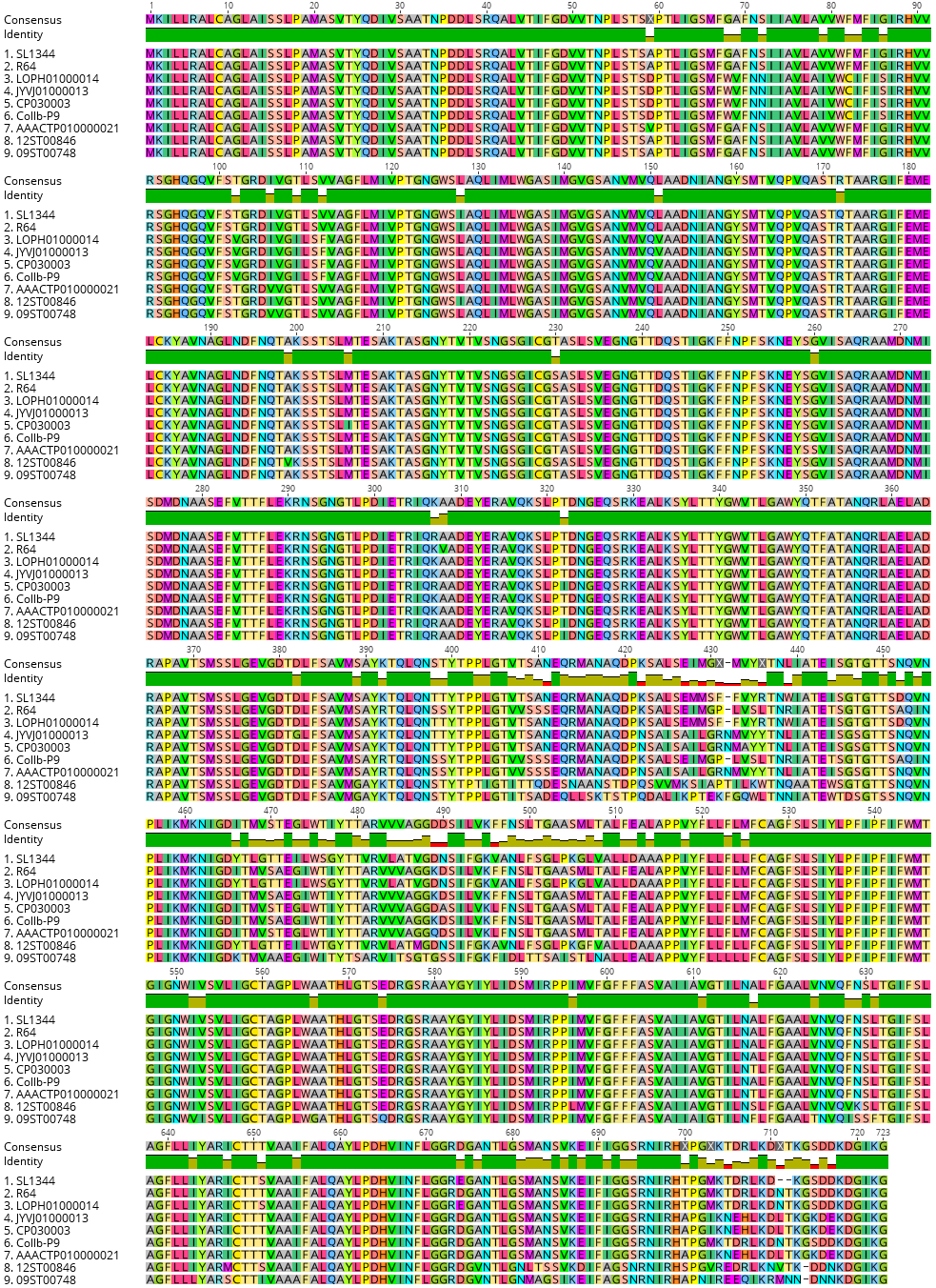

Supplement: Supplementary file 7 — Additional file 7. Figure S4. Alignment of TraY protein sequences from nine IncI1 plasmids. [file 12866_2021_2151_MOESM7_ESM.tif]
