# Supplementary material for: Characterisation of IncI1 plasmids associated with change of phage type in isolates of Salmonella enterica serovar Typhimurium
Source: BMC Microbiol. 2021 Mar 27;21:92. doi: 10.1186/s12866-021-02151-z (PMC8004404; doi:10.1186/s12866-021-02151-z)
Supplement: Supplementary file 8 — Additional file 8. Text S2. Search results for variants of ExcA proteins in the NCBI database. [file 12866_2021_2151_MOESM8_ESM.docx]

**Search results for variants of ExcA proteins in the NCBI database**

BLASTp with R64 ExcA yielded many hits at 100% identity across *E. coli, Salmonella, S. sonnei, Klebsiella* and *Citrobacter.* TraY was either 100% identity, or very close, to R64 or ColIb-P9. The plasmids were either Delta or Col IncI1 type with full or partial Delta or Col gene sets. *S.* Hadar plasmid pPIR00503 (GenBank Acc. No. PKPF01000003) had a combination of Delta and Col genes. It had Delta genes *ydbA* to *ydfA* including the phage inhibiting gene *ibfA* as well as Col genes *ycbA* to the *rfsF* site including the ColIb-P9-specific phage inhibiting gene *ibfA*.

BLASTp with ColIb-P9 ExcA yielded many hits at 100% identity across *E. coli, Salmonella* and *S. sonnei.* TraY was nearly always 100% identity or close to ColIb-P9 with a few close variants of R64 TraY. Most plasmids were Col type with a few Delta type. The *S.* Typhimurium plasmid p9134 (GenBank Acc. No. NC_023275) was found to have a full set of Delta genes as well as many Col genes and appeared to be a Delta/Col recombinant. BLASTp with 12ST00846 ExcA yielded hits at 100% identity across *E. coli, Salmonella* and *S. sonnei.* The TraY was always the 12ST00846 variant or close to it. Most plasmids were the Col type but a few were the Delta type. BLASTp with 09ST00748 ExcA yielded hits at 100% identity across *E. coli, Salmonella* and *S. sonnei.* The TraY was always the 09ST00748 variant or close to it. Most plasmids were the Col type but a few were the Delta type. BLASTp with SL1344 ExcA yielded hits at 100% identity across *E. coli, Salmonella, S. sonnei* and *Klebsiella.* The TraY was mostly the same as or a close variant of SL1344 TraY which has 2 gaps relative to R64 TraY. A few plasmids had TraY closer to ColIb-P9 TraY than to R64 TraY without the 2 gaps. These TraY appeared to be recombinants between ColIb-P9 and SL1344 TraY protein. BLASTp with GenBank Acc. No. JYVJ01000013 ExcA yielded hits at 100% identity mostly across *E. coli* and *Salmonella.* There were three distinct but related TraY proteins. One was the JYVJ01000013 TraY with 678/723 identity to R64 TraY with one gap. Another was the *S.* Brandenburg GenBank Acc. No. CP030003 TraY with 669/723 identity to R64 TraY and the same gap as JYVJ01000013 TraY and the third the *Salmonella* GenBank Acc. No. AAACTP010000021 TraY with 689/723 identity to R64 TraY with the same gap.
