# Supplementary material for: Characterisation of IncI1 plasmids associated with change of phage type in isolates of Salmonella enterica serovar Typhimurium
Source: BMC Microbiol. 2021 Mar 27;21:92. doi: 10.1186/s12866-021-02151-z (PMC8004404; doi:10.1186/s12866-021-02151-z)
Supplement: Supplementary file 9 — Additional file 9. Text S3. Search results for the rfsF to yfcA 9177 bp sequences in plasmids of sequenced isolates in the NCBI database. [file 12866_2021_2151_MOESM9_ESM.docx]

**Search results for the *rfsF* to *yfcA* 9177 bp sequences in plasmids of sequenced isolates in the NCBI database.**

The plasmids in Cluster A had almost total identity with R64 except 03ST04727 being a Col plasmid had the same SNP in the *rfsF* site as seen for ColIb-P9. P212_15 and the other two U307 plasmids in Cluster B1 were all nearly the same with around 37 SNPs different from R64 and five SNPs in the *rfsF* site. Except for 09ST05380 the plasmids in Cluster B2 all had matching sequence from *rfsF* to *yfdB* with 17 SNPs including one in the *rfsF* site but *yfcA* was replaced with another sequence. The plasmid in 08ST05125 was 28 SNPs different from R64 but the *yedA* gene was replaced by another sequence. Three plasmids in Cluster C, 07ST03750, 08ST06126 and 11ST07272 were different from R64 by 151 SNPs mostly scattered throughout the sequence. 09ST01733 in Cluster C had an additional 29 SNPs confined to a small part of the sequence. 09ST01531 also in Cluster C was 164 SNPs different from R64 but the sequence between *yefA* and *parB* was replaced by a different sequence including two partitioning genes which were different from the *parA* and *parB* genes in both R64 and R621a. The partitioning genes in 09ST01531 were found in more than 132 isolates of *Enterobacteriaceae* by BLASTp. The plasmid in 12ST03486 was missing all sequence from *rfsF* to *parB.* There were several replacement genes including one identified as a partitioning gene but it was not like other such genes and was found in only a few isolates by BLASTp. The two closely related plasmids with no RDs, 12ST00846 and 10ST01093 both had the *yedA* gene replaced by two genes which also occurred in the same location in 10ST00233 (see below). There were 79 and 33 SNPs respectively in the sequence before the replacement genes and 188 SNPs in the sequence after. The plasmid from 01ST04081 with RD3 only had two genes substituting for the *yedA* gene. There were 39 SNPs in the sequence before the replacement genes and 153 in the sequence after. The plasmid from 10ST03440 had 169 SNPs overall. It was missing the first 17 bp of the *rfsF* site and there were two insertion sequences located in the *impB* gene. The plasmid from 10ST00233 was missing all but the last six bp of the *rfsF* site and had four genes replacing the *yedA* gene. The last two of these genes were also found in the same location in 12ST00846 and 10ST01093. There were 36 SNPs in the sequence before the replacement genes and 216 after. In summary, the *rfsF* to *yfcA* region showed considerable variation in both gene composition and DNA sequence among the plasmids from the sequenced isolates. Plasmids which clustered closely in the phylogenetic tree mostly had the same or nearly the same sequence for the region and the sequences for each cluster and the singletons were all distinctly different from each other giving support to the conclusions drawn from the tree.
