# Supplementary material for: Characterisation of IncI1 plasmids associated with change of phage type in isolates of Salmonella enterica serovar Typhimurium
Source: BMC Microbiol. 2021 Mar 27;21:92. doi: 10.1186/s12866-021-02151-z (PMC8004404; doi:10.1186/s12866-021-02151-z)
Supplement: Supplementary file 10 — Additional file 10. Text S4. Search results for variants of YdaA proteins in the NCBI database. [file 12866_2021_2151_MOESM10_ESM.docx]

**S4 Text. Search results for variants of YdaA proteins in the NCBI database**

BLASTing in the NCBI database with the protein sequence for the Cluster A YdaA variant from 09ST00748 produced eight listings of *E. coli* with identical YdaA protein in IncI1 plasmids and with very high identity to 09ST00748 plasmid over the Delta gene set as well as most of the shared genes. There were three closely related isolates from USA with very high coverage including most of the antimicrobial and metals resistance insert. There was one isolate each from Australia, China and Germany. There were two isolates from turkey samples in the USA which had missing Delta genes from the *ydfA* gene onwards like 11ST04232. There were seven listings of closely related DT104 *S*. Typhimurium isolates which had all of the Delta gene set at high identity but located in pSLT plasmids as noted previously [17]. There were numbers of other examples of *E*. *coli* plasmids with all or most of the Delta gene set in other types of plasmid such as IncFIB.

BLASTing with the protein sequence for the YdaA from R64 identified only two IncI1 plasmids with very close identity and coverage to R64 but not to so close to members of Cluster C with RD2 only. Both were located in *S.* Heidelberg isolates from the USA, GenBank Acc. No. AMMX0100000000 isolated in 1982 and GenBank Acc. No. AMLR0100000000 isolated in 1987. BLASTing with the YdaA variant from 08ST00576 in Cluster B2 identified several IncI1 plasmids with a complete set of Delta genes in *Salmonella* isolates but none had shared genes close to Cluster B2 members although one had very high identity to the shared gene set for 09ST00748 even though the YdaA variant was from 08ST00576. Again there were numbers of plasmids in *E.coli* isolates with the complete Delta gene set or stopping around the *ydfB* gene but they were not IncI1 plasmids. Some were IncF or IncFIB plasmids. Results were similar for BLASTing with the YdaA variant from 12ST00846 (no RD) although there were more isolates identified mostly belonging to *E.coli*. Nearly all the plasmids investigated had the complete Delta gene set. Some were IncI1 plasmids but none was close overall to any members of clusters in this study.
